# Interactivity: A Potential Determinant of Learning by Preparing to Teach and Teaching

**DOI:** 10.3389/fpsyg.2018.02755

**Published:** 2019-01-11

**Authors:** Keiichi Kobayashi

**Affiliations:** Faculty of Education, Shizuoka University, Shizuoka, Japan

**Keywords:** leaning by teaching, learning by preparing to teach, interactivity, explaining to others, explaining to oneself, direct teaching, indirect teaching

## Abstract

It has been suggested that preparing to teach and teaching are conditionally effective in enhancing one’s own learning. This paper focuses on interactivity – the level of teacher–student interaction in expected or actual teaching – as the potential key to understanding and controlling the variability in the effectiveness of learning by preparing to teach and teaching. By summarizing and reanalyzing the results of previous studies, I suggest that the learning benefits of studying with the expectation of direct teaching (i.e., teaching a student face-to-face) are greater than those of studying with the expectation of indirect teaching (i.e., teaching a student indirectly by creating a lecture video, providing written explanations, or using other means) and that learning by direct teaching surpasses learning by explaining to oneself or indirect teaching at least after preparing to do so. Next, three candidate explanations for the impact of interactivity are discussed: the advantages of asking and answering questions, obtaining additional information about and from one’s student, and enhancing one’s motivation to process learning material deeply while preparing to teach and teaching. Finally, I conclude with the remaining questions and directions for future research.

## Introduction

In academic situations, such as peer tutoring and small group activities, students have a rich opportunity to learn by preparing to teach (or explain) the contents of learning material to others (e.g., tutees, other group members) and teaching others. It is suggested that both preparing to teach and teaching stimulate teacher-role students – namely, students playing the role of tutor or explainer in a learning activity – to process learning material generatively and constructively – for example, selecting, elaborating, and organizing important information from the learning material, integrating newly acquired information with prior knowledge, and reflecting on their own comprehension – and thereby enhance their learning (Annis, 1983; Benware and Deci, 1984; Coleman et al., 1997; van Blankenstein et al., 2011; Fiorella and Mayer, 2013, 2014; Matsuda et al., 2013; Hoogerheide et al., 2014; Nestojko et al., 2014; Daou et al., 2016a; for reviews, see Webb, 1991; Fiorella and Mayer, 2015; Duran, 2017).

However, this does not mean that learning by preparing to teach and teaching is unconditionally effective. Indeed, there exists a body of research showing that, at least under certain conditions, students scarcely, if at all, benefitted from studying with the expectation of teaching others (Ehly et al., 1987; Renkl, 1995; Ito and Kakihana, 2009; Hoogerheide et al., 2016) and teaching (Bargh and Schul, 1980; Ito and Kakihana, 2009; Hoogerheide et al., 2016; Koh et al., 2018). Roscoe and Chi (2007) also suggested that, regardless of grade level and subject domain, peer tutoring may have a limited effect on tutors’ learning because they often process learning material superficially during the tutoring interaction, without inferring, elaborating, or monitoring. It is now crucial to understand not only whether and why learning by preparing to teach and teaching is effective but also what substantially affects its effectiveness.

By highlighting the role of interactivity, this paper sheds light on the variability in the learning effects of preparing to teach and teaching. *Interactivity* here refers to the extent to which teacher-role students expect to or do interact with their students while teaching. The teacher–student interaction includes providing explanations, adding supplementary explanations, asking and answering questions, and giving and receiving feedback. In this paper, interactivity is classified into three levels: non-interactive (i.e., explaining to oneself), indirect teaching, and direct teaching. *Indirect teaching* is to teach or explain to a student indirectly by creating a lecture video, providing written explanations, or using other means. *Direct teaching* is defined as teaching or explaining to a student in a face-to-face manner. Although my classification of interactivity builds on Plötzner et al. (1999) and Kobayashi (2018), the classification in these studies focused on explaining to oneself, others, and each other (Plötzner et al., 1999) or excluded non-interactive from consideration (Kobayashi, 2018; see Table 1). The classification in this paper enables the adoption of two approaches to the impact of interactivity: direct teaching versus non-interactive and direct versus indirect teaching (see below for further details).

**Table 1:** Classification of interactivity in Plötzner et al. (1999), Kobayashi (2018), and this paper.

Explanation and teaching	Plötzner et al. (1999)	Kobayashi (2018)	This paper
Explaining to oneself	Level 1	–	Non-interactive
Explaining to an imaginary or anonymous student (e.g., creating a lecture video, providing written explanations)	Level 2	Non-interactive teaching	Indirect teaching
Explaining to a passive and anonymous student who just listens	Level 3	–	–
Explaining to a student of one’s acquaintance who responds in a constrained way; teaching a student face-to-face, without asking or answering questions	Level 4	Interactive teaching	Direct teaching
Teaching a student face-to-face, asking and answering questions	–	Interactive teaching	Direct teaching
Mutually explaining to each other	Level 5	–	–


The role of interactivity in learning by teaching, particularly learning by preparing to teach, has received relatively little attention from researchers. Yet, there is good reason to believe that interactivity substantially affects the learning effects of preparing to teach and teaching. In the subsequent sections, I first review related work, including the reanalysis of data from Kobayashi’s (2018) meta-analytic study, to substantiate this contention. Next, I discuss some candidate explanations for the impact of interactivity. Finally, remaining questions and future directions are considered.

## The Impact of Interactivity on Learning by Preparing to Teach and Teaching

To evaluate the impact of interactivity on learning by preparing to teach and teaching, I adopt the following two approaches. One approach – direct teaching versus non-interactive – is to determine whether and how explaining to others face-to-face differs from explaining to oneself (including thinking aloud) in learning performance. Explaining to oneself is non-interactive in that generated explanations are not directed at anyone, except for experimenters, whereas explaining to others face-to-face is on a higher level of interactivity (see Table 1). Therefore, the difference between learning by explaining to others face-to-face and oneself can be interpreted to indicate the impact of interactivity.

Several studies have investigated the learning effects of explaining to others face-to-face versus oneself. For example, Coleman et al. (1997) had students study learning material with the expectation of explaining its contents to their partners face-to-face or to themselves and then actually do so. On near and far transfer tasks, those who explained to their partners outperformed those who explained to themselves. Similar results were reported by Rittle-Johnson et al. (2008) and Chase et al. (2009). In Rittle-Johnson’s et al. (2008) study, 4- and 5-year-olds, who had been taught correct solutions to multiple classification problems in advance, performed better on a transfer task after they explained the correct solutions to their mothers than they did after they explained the solutions to themselves. Using a computer-based learning environment, Chase et al. (2009) found that, other things being equal, students who were presented an interactive computer character as a teachable agent learned more deeply by teaching the character than those who were presented the character as their avatars. By contrast, in a study by Bargh and Schul (1980), students who taught another student face-to-face did not differ in learning outcomes from those who verbalized their thoughts while performing a learning task. Roscoe and Chi (2008) also found that explaining to oneself led to a deeper understanding than tutoring face-to-face.

Notably, the studies with inconsistent findings differed in whether the participants could or did prepare to explain before they provided explanations. In the studies by Coleman et al. (1997); Rittle-Johnson et al. (2008), and Chase et al. (2009), the participants were instructed to prepare for explanation or could expect that they would explain to others face-to-face or explain to themselves afterward. On the other hand, Bargh and Schul (1980) and Roscoe and Chi (2008) did not inform the participants of the subsequent teaching or explanation at all. Taken together, these findings suggest that students learn by explaining to others face-to-face more than explaining to themselves only after they study with the expectation of doing so. Preparing for face-to-face explanation may be a prerequisite for learning effectively by explaining to others face-to-face.

Another approach – direct versus indirect teaching – is to compare the learning effects of (preparing for and/or actual) direct teaching with those of indirect teaching. Both direct and indirect teaching are directed at others, but direct teaching is higher in interactivity than indirect teaching. Thus, the comparison between learning by direct and indirect teaching would provide useful information on the impact of interactivity. To my knowledge, only two experimental studies examined the learning effects of direct versus indirect teaching. Roscoe and Chi (2008) found that tutoring face-to-face outperformed explaining on video. Similarly, Ito and Kakihana (2009) found that students who explained the contents of learning material to another student face-to-face (after they studied the learning material with the expectation of doing so) performed better in memory and comprehension of the learning material than those who provided videotaped explanations. Merely studying with the expectation of explaining face-to-face did not differ from studying with the expectation of explaining on video.

The original and reanalyzed results of Kobayashi (2018), who meta-analytically examined the impact of interactivity as a moderator on learning by preparing to teach and teaching, would compensate for the paucity of experimental research adopting the second approach. In the original meta-analyses, there were 28 groups studying with versus without teaching expectancy and 16 groups studying with teaching expectancy and subsequent teaching versus studying without teaching expectancy. My reanalysis of Kobayashi (2018) included additional data from Fiorella and Mayer (2014) and Hoogerheide et al. (2016) to estimate the learning benefits of indirect teaching after studying without teaching expectancy. The results of the moderator analyses are shown in Table 2. The weighted mean effect sizes (Hedges’s *g*s) for studying with versus without teaching expectancy were larger when direct teaching was expected, *g* = 0.50, than when indirect teaching was expected, *g* = 0.27, *Q*_B_(1) = 6.51, *p* < 0.05. Direct teaching after studying with direct teaching expectancy (relative to merely studying without teaching expectancy) had a larger beneficial effect, *g* = 0.84, than indirect teaching after studying with indirect teaching expectancy, *g* = 0.48, which in turn exceeded indirect teaching after studying without teaching expectancy, *g* = 0.23, *Q*_B_(2) = 118.79, *p* < 0.001.

**Table 2:** Impact of interactivity as a moderator on learning by preparing to teach and teaching.

Group comparison (versus control)	*k*	*g*	95% CI	*Q*_w_	*Q*_b_
**Effects of preparing to teach^a^**					
Studying with indirect teaching expectancy	18	0.27	[0.17, 0.37]	39.12^∗∗∗^	6.51^∗^
Studying with direct teaching expectancy	10	0.50	[0.36, 0.64]	74.39^∗∗∗^	
**Combined effects of preparing to teach and teaching^b^**					
Indirect teaching after studying without teaching expectancy	2^c^	0.23	[0.11, 0.35]	0.05	118.79^∗∗∗^
Indirect teaching after studying with indirect teaching expectancy	12	0.48	[0.43, 0.53]	117.22^∗∗∗^	
Direct teaching after studying with direct teaching expectancy	4	0.84	[0.75, 0.93]	44.29^∗∗∗^	


*The random-effects model was used. k = number of group comparisons. g = weighted mean effect size (Hedges’s g). CI = confidence interval. Q_w_ = within-group homogeneity statistic. Q_b_ = between-group homogeneity statistic. ^a^The results of Kobayashi’s (2018) moderator analysis (non-interactive versus interactive teaching expectancy). ^b^Data from Kobayashi (2018) were reanalyzed, including additional data from Fiorella and Mayer (2014) and Hoogerheide et al. (2016). ^c^Fiorella and Mayer (2014), experiment 2, expect test – teach group (*n* = 27) versus expect test – no teach group (n = 24, g = 0.21); Hoogerheide et al. (2016), experiment 1, test intention – explain in writing group (n = 33) versus test intention – restudy group (n = 29, g = 0.24). ^∗^p < 0.05. ^∗∗∗^p < 0.001.*

In summary, the currently available evidence, while still inadequate and subject to some exceptions, suggests that a higher level of interactivity in expected and actual teaching substantially increases learning by preparing to teach and subsequent teaching. More specifically, as compared to studying with the expectation of indirect teaching, studying with the expectation of direct teaching may be beneficial to learning. Students may also learn by direct teaching more than explaining to oneself and indirect teaching, at least when they study with the expectation of doing so beforehand.

## Candidate Explanations for the Impact of Interactivity

Why does interactivity affect learning by preparing to teach and teaching? The literature suggests at least three explanations. The first explanation is that, unlike explaining to oneself and indirect teaching, direct teaching has the additional advantage of asking and answering questions, if necessary, during the teacher–student interaction (Roscoe and Chi, 2007; Duran, 2017). The generation of good questions requires deep processing of learning material and metacognitive monitoring of one’s own knowledge and comprehension; indeed, it has been shown that teaching students questioning skills increases their learning (e.g., King, 1992; Rosenshine et al., 1996). Thus, according to Roscoe and Chi (2007), students who teach directly may benefit from generating questions for their students if the processes entail reflective knowledge building – organizing and inferring from learning material, integrating new information with prior knowledge, and discovering and filling a gap in their own comprehension. Moreover, questions from one’s student may stimulate reflective knowledge building by eliciting the process of self-examination (Roscoe and Chi, 2008; Roscoe, 2014). However, some studies revealed that merely explaining to others without asking or answering questions was more effective than explaining to oneself (Coleman et al., 1997; Rittle-Johnson et al., 2008) and explaining on video (Ito and Kakihana, 2009), suggesting that the learning benefits of direct teaching are not attributed solely to the advantage of questioning. This explanation, even if correct, would be limited to situations in which teacher–student interaction includes asking and answering questions.

The second explanation states that even when questioning is not allowed, direct teaching gives teacher-role students an opportunity to obtain additional information about and from their students, which in turn contributes positively to their learning (Ito and Kakihana, 2009). For instance, there is some evidence that being informed about others’ knowledge and understanding enhances learning by explaining to others (e.g., Zufferey et al., 2010; Ray et al., 2013). Okita and Schwartz (2013) and Okita et al. (2013) also found that tutors who taught their tutees and then observed their tutees taking a test surpassed those who repeatedly taught their tutees in comprehension of learning material. However, these studies did not assume that, during interaction, teacher-role students accurately infer their students’ knowledge and understanding without external support and skillfully use information from their students. As Okita and Schwartz (2013) and Okita et al. (2013) pointed out, direct feedback from one’s student is not always clear or straightforward, making it difficult to immediately and fully comprehend what the feedback informs about one’s explanations. It is questionable whether teacher-role students, who have limited teaching experience and knowledge about subject matter content, effectively deal with information about and from their students and thereby reflect on and deepen their understanding while actively interacting with their students.

The third explanation emphasizes the motivational aspect of interactivity. In this view, expected and actual direct teaching motivates teacher-role students to process learning material deeply (Coleman et al., 1997; Rittle-Johnson et al., 2008). For example, on the assumption that the processes of teaching others satisfy essential preconditions for intrinsic motivation, that is, one’s needs to determine for oneself and influence one’s environment meaningfully, Benware and Deci (1984) posited that the expectation of direct teaching enhances students’ intrinsic motivation to study learning material, thereby leading to deep learning. Chase et al. (2009) suggested that the *protégé effect*, which refers to a phenomenon that people learn more effortfully for a teachable agent than for themselves, constitutes the advantage of learning by direct teaching. Direct teaching may also provide an opportunity to raise one’s own self-efficacy and self-esteem (Rienovita et al., 2018). The motivational explanation is appealing in that, unlike the first and second explanations, it accounts for the impact of interactivity on learning by preparing to teach as well as teaching. Unfortunately, existing evidence regarding the motivational effects of interactivity is limited and mixed. Benware and Deci (1984) found that, as they predicted, the expectation of direct teaching increased intrinsic motivation to study learning material more than the expectation of taking a test, whereas other studies (Renkl, 1995; Daou et al., 2016a,b, 2018) failed to replicate the results. In line with the motivational explanation, Chase et al. (2009) indicated that students who taught the teachable agent spent more time engaging in learning activities than those who taught their avatars. Rienovita et al. (2018) found that teaching other students in an interactive peer learning activity increased teacher-role students’ self-esteem but decreased their self-efficacy.

In sum, to date, there is no single satisfactory explanation for why interactivity affects learning by preparing to teach and teaching. This finding is not surprising, considering that the impact of interactivity has received scant research attention. The three explanations are not mutually exclusive, and therefore, some of the explanations may account for the impact of interactivity in concert. In any case, more research is needed to understand underlying mechanisms.

## Implications for Future Research

This paper is the first to review a relatively broad range of evidence and suggest that interactivity is the potential key to understanding and controlling the variability in the learning effects of preparing to teach and teaching. Nevertheless, available and solid evidence regarding the impact of interactivity is still limited, leaving some important questions unanswered.

The first question concerns the influence of direct or indirect teaching expectancy on learning by preparing to teach and subsequent teaching. The literature review suggests that effective learning by direct teaching requires preparing to teach others directly. Additionally, the findings that the learning benefits of indirect teaching were greater after studying with indirect teaching expectancy (*g* = 0.48) than after studying without teaching expectancy (*g* = 0.23) implies that preparing for indirect teaching may make a meaningful contribution to learning by indirect teaching. However, no research has investigated the learning effects of direct teaching after studying with the expectation of indirect teaching or of indirect teaching after studying with the expectation of direct teaching, thus making it difficult to disentangle the impact of direct or indirect teaching expectancy from that of teaching expectancy *per se*. Researchers should determine whether the level of interactivity in expected teaching affects the combined effects of preparing to teach and subsequent teaching, and if so, how and why.

The second question is whether and how learning by preparing to teach and teaching differs according to modes of interacting (or expecting to interact) with others. Although prior work has focused on face-to-face teaching and one-way explanation, these do not cover all possible modes of interaction. For example, in computer-mediated learning situations, teacher–student interaction may proceed via the asynchronous exchange of written messages. It is also possible to image a learning scenario in which teacher-role students teach their students by telephone, smartphone, or video telephony. Each mode of interaction is distinguished by some factors, such as modality (e.g., oral, written), the visual and physical presence of teacher-role students and their students, and interactional immediacy. A systematic investigation of the effects of different interaction modes will assist in identifying which aspects of interactivity are crucial to the improvement of learning by preparing to teach and teaching.

Third, my argument in this paper relied mainly on the findings of studies conducted in artificial experimental settings. Thus, some caution is needed when estimating the impact of interactivity outside the lab. For methodological reasons, the experimental settings did not always reflect real academic situations in which students may learn by preparing to teach and teaching. In such situations, teacher-role students may have more knowledge about their students, a heavier responsibility to foster their students’ learning, and repeated opportunities for teaching, regardless of whether they teach their students directly or indirectly. Whether interactivity plays a role in learning by preparing to teach and teaching within the authentic context of educational practice is an important question that needs to be addressed.

Finally, it will be worthwhile to investigate the impact of interactivity from a developmental perspective. There is a growing body of evidence that the acts and capability of teaching emerge in early stages of development and are gradually refined (e.g., Strauss and Ziv, 2012; Calero et al., 2015), suggesting that even young children have opportunities to learn by preparing to teach and teaching. More importantly, some studies have provided evidence that preschool and elementary school children benefit from tutoring or explaining to others face-to-face (e.g., Cohen et al., 1982; Rittle-Johnson et al., 2008) and creating a lecture video for other children in classroom settings (Muis et al., 2016). But still, it remains unclear whether and how the impact of interactivity varies across development. Given that developmentally earlier forms of teaching usually entail face-to-face interaction (see e.g., Strauss and Ziv, 2012) and that composing a message for a potential audience is cognitively complex and demanding (e.g., Bereiter and Scardamalia, 1987; Kuhn and Udell, 2003), it may be too difficult for younger children to learn by indirect teaching without help. Conversely, for older children and adults, interactivity may have a relatively weak impact. Future research could examine these possibilities.

## Author Contributions

The author designed the paper, analyzed the literature, and drafted the manuscript.

## Conflict of Interest Statement

The authors declare that the research was conducted in the absence of any commercial or financial relationships that could be construed as a potential conflict of interest.
